# United States Government War-Savings Stamps

**Published:** 1918-03

**Authors:** 


### UNITED STATES GOVERNMENT WAR-SAVINGS STAMPS

In offering "*War-Savings Stamps*" to the public the United States Government has made immediately available for every man, woman, and child in the country a profitable, simple, and secure investment.

WHAT THEY ARE.—*War-Savings Stamps* are the answer of a great democracy to the demand for a democratic form of government security. They are "little baby bonds." Like Liberty bonds, they have behind them the entire resources of the Government and people of the United States. They have the additional advantage that they steadily increase in value from the date of purchase until the date of maturity, and this increase is guaranteed by the Government. These stamps are issued in two denominations, the 25-cent stamp and the $5 stamp.

For the convenience of investors a "*Thrift Card*" is furnished to all purchasers of 25-cent stamps. This card has spaces for 16 stamps. When all the spaces have been filled the *Thrift Card* may be exchanged for a $5 stamp at post offices, banks, or other authorized agencies by adding 12 cents in cash prior to February 1, 1918, and 1 cent additional each month thereafter.

Those who prefer may buy a $5 stamp outright. These will be on sale from December 3, 1917, until January 31, 1918, for $4.12. They automatically increase in value a cent a month every month thereafter until January 1, 1923, when the United States will pay $5 at any post office or at the Treasury in Washington for each stamp affixed to a *War-Savings Certificate.*

When you purchase a $5 stamp, you must attach it to an engraved folder known as a "*War-Savings Certificate*" which bears the name of the purchaser and can be cashed only by the person whose name appears upon the certificate, except in case of death or disability. This certificate contains 20 spaces. If these are all filled with *War-Savings Stamps* between December 3, 1917, and January 31, 1918, the cost to the purchaser will be $82.40, and on January 1, 1923, the Government will pay the owner of the certificate $100—a net profit to the holder of $17.60. This is based on an interest rate of 4% compounded quarterly from January 2, 1918. The amount of *War-Savings Stamps* sold to any one person at any one time shall not exceed $100 (maturity value), and no person may hold such stamps or *War-Savings Certificates* to an aggregate amount exceeding $1,000 (maturity value).

If the holder of a *War-Savings Certificate* finds it necessary to realize cash on it before maturity, he may at any time after January 2, 1918, upon giving 10 days' written notice to any money-order post office, receive for each stamp affixed to his certificate the amount paid therefor plus 1 cent for each calendar month after the month of purchase of each stamp. A registered certificate may be redeemed, however, only at the post office where registered.

In other words, the plan is simple, straightforward, and certain. The holder of the certificates can not lose and is certain to gain. He is buying the safest security in the world in the most convenient form in which the security of a great Government has ever been offered to its people.

*Why You Should Buy Them.*—The main reason for the purchase of *War-Savings Stamps* is because your country is at war. Your country needs every penny which every man, woman, and child can save and lend, in order to feed, clothe, arm, and equip the soldiers and sailors of America and to win this righteous war in defense of American honor and the cause of democracy throughout the world.

If we are to win the war, we must win it as a united people. The savings of every man, woman, and child are necessary if we are to hasten the victorious ending of the war. *War Savers are Life Savers.*

A single strand in the cables which uphold the great Brooklyn Suspension Bridge is not very strong, but thousands of these strands bound together uphold one of the great thoroughfares of the world.

When our fathers and sons and brothers were called by our Country to take up arms in her defense, you did not hear an individual soldier refuse to serve because his service alone would not win the war. Each man was ready to do his part. The great army thus formed is going forward to face the fire of battle and to risk everything for the safety and security of our homes and our families, and for the very existence of our Country.

These are the men for whom you are asked to save and lend your dollars.

A Country worth *fighting* for is a Country worth *saving* for.

To save money is to save life.

Buy War-Savings Stamps at post offices, banks, trust companies, or other authorized agencies, and strike a blow for our Country.

